# Discovery of Spilanthol Endoperoxide as a Redox Natural Compound Active against Mammalian Prx3 and *Chlamydia* *trachomatis* Infection

**DOI:** 10.3390/antiox9121220

**Published:** 2020-12-03

**Authors:** Rosine Dushime, Yunhuang Zhu, Hanzhi Wu, Daniel Saez, Kirtikar Shukla, Heather Brown-Harding, Maique W. Biavatti, Kimberly J. Nelson, Leslie B. Poole, William T. Lowther, Paul B. Jones, Cristina M. Furdui, Allen W. Tsang

**Affiliations:** 1 Department of Internal Medicine, Section on Molecular Medicine, Wake Forest School of Medicine, Winston-Salem, NC 27157, USA; Rosine.Dushime@cmcmaterials.com (R.D.); hwu@wakehealth.edu (H.W.); danielsaez713@gmail.com (D.S.); kshukla@wakehealth.edu (K.S.); 2 Department of Chemistry, Wake Forest University, Winston-Salem, NC 27109, USA; zhuy216@wfu.edu (Y.Z.); jonespb@wfu.edu (P.B.J.); 3 Department of Biology, Wake Forest University, Winston-Salem, NC 27157, USA; brownhh@wfu.edu; 4 Centro de Ciências da Saúde, Departamento de Ciências Farmacêuticas, Bloco J/K, Universidade Federal de Santa Catarina, Florianópolis 88040-900, SC, Brazil; maique.biavatti@ufsc.br; 5 Department of Biochemistry, Wake Forest School of Medicine, Winston-Salem, NC 27157, USA; kinelson@wakehealth.edu (K.J.N.); lbpoole@wakehealth.edu (L.B.P.); tlowther@wakehealth.edu (W.T.L.)

**Keywords:** chlamydia trachomatis, spilanthol, spilanthol endoperoxide, dioxacmellamide, mitochondria, peroxiredoxin, Prx3

## Abstract

*Chlamydia trachomatis* (Ct) is a bacterial intracellular pathogen responsible for a plethora of diseases ranging from blindness to pelvic inflammatory diseases and cervical cancer. Although this disease is effectively treated with antibiotics, concerns for development of resistance prompt the need for new low-cost treatments. Here we report the activity of spilanthol (SPL), a natural compound with demonstrated anti-inflammatory properties, against Ct infections. Using chemical probes selective for imaging mitochondrial protein sulfenylation and complementary assays, we identify an increase in mitochondrial oxidative state by SPL as the underlying mechanism leading to disruption of host cell F-actin cytoskeletal organization and inhibition of chlamydial infection. The peroxidation product of SPL (SPL endoperoxide, SPL^E^), envisioned to be the active compound in the cellular milieu, was chemically synthesized and showed more potent anti-chlamydial activity. Comparison of SPL and SPL^E^ reactivity with mammalian peroxiredoxins, demonstrated preferred reactivity of SPL^E^ with Prx3, and virtual lack of SPL reaction with any of the reduced Prx isoforms investigated. Cumulatively, these findings support the function of SPL as a pro-drug, which is converted to SPL^E^ in the cellular milieu leading to inhibition of Prx3, increased mitochondrial oxidation and disruption of F-actin network, and inhibition of Ct infection.

## 1. Introduction

*Chlamydia trachomatis* (Ct) is one of the most prevalent sexually transmitted infections in the world with close to 130 million new cases arising each year [1]. Ct infections are responsible for a host of diseases including pelvic inflammatory disease, infertility, ectopic pregnancy disease [2], and causing a potential 200–300% increased chance of HIV contraction [3]. Ct infection has also been linked to an increased risk of cervical cancer [4,5]. Although the molecular mechanisms underlying this activity have not been fully elucidated, we reported in earlier studies Ct upregulation of host cell epidermal growth factor receptor (EGFR) signaling, a hallmark of cervical cancer [6]. 

Chlamydial infection is characterized by a complex intracellular developmental cycle, which has not yet been exploited for the discovery of new therapies against Ct infections. Briefly, these pathogenic bacteria exist in three distinct morphologies. The infectious morphology of Ct is the elementary body (EB), which is largely metabolically inactive and serves to attach to host cell membranes and initiate infection [7]. Following host cell infection, the EBs differentiate into a metabolically active, intracellular reticulate body (RB) [7]. Ct morphologies exist in a protective vacuole termed the inclusion, which is supported by the re-organization of host cell cytoskeletal protein F-actin into a dynamic ring to stabilize and protect the inclusion [7]. Inside the inclusion, the RBs asynchronously either 1) replicate into more RBs enlarging the inclusion, 2) differentiate back to the EB state, which then exits the cells through lysis or extrusion [8] leading to infection of neighboring cells, or 3) differentiate to an AB state (aberrant bodies) [7]. It has been documented that stressors such as nutrient deprivation and antibiotic challenge can lead to inhibition of the developmental cycle and a persistent chlamydial phenotype dominated by the presence of ABs [9,10]. 

Given these concerns, the lack of an FDA-approved vaccine to prevent Ct infections [11], and recent reports showing the possibility for increased resistance to doxycycline and azithromycin antibiotics, the standard of care for Ct treatment in the clinic [12], there is an immediate need for improved treatment methods. Additionally, due to the global prevalence of Ct infections, the new therapeutics must be low cost and easily manufactured. Our previous studies have demonstrated the application of anti-EGFR (host cell Epidermal Growth Factor Receptor) antibodies like Cetuximab, which binds to the extracellular domain of EGFR, or kinase inhibitors like Erlotinib for treatment of Ct infections [6]. However, as Cetuximab and Erlotinib are typically used for cancer treatment, they are prohibitively expensive limiting the translation to the clinic for treatment of Ct infection on a global scale. To achieve a higher therapeutic index and lower cost, we have initiated studies with natural compounds, which historically have better safety-efficacy profiles, have superior chemical diversity and complexity, offer the opportunity for affordable drug development, and have been used for thousands of years in traditional medicine. In fact, ~50% of all current drugs have originated from studies using plant compounds, and the World Health Organization estimates that ~80% of the world population uses traditional medicine for primary healthcare needs including treatment of infections [13].

Recently, we reported the unexpected activity of sulforaphane, a compound with demonstrated anti-inflammatory properties, as a promoter of Ct infections [14]. In the studies presented here, we investigated the effects of spilanthol (SPL), another natural product with reported anti-inflammatory activity [15,16], in Ct infections. Both compounds have demonstrated reactivity with protein reactive cysteine residues modulating cellular processes [14,17]. SPL is an alkamide found in several Heliantheae species, the third-largest tribe in the sunflower family, *Acmella ciliata*, *Spilanthes acmella* and other SPL rich plants used as traditional remedies throughout the world [18,19]. The leaves and flowers of these plants have sensory properties (pungency, tingling, numbing, mouth-watering) making them a popular spice and ingredient in several Brazilian dishes as well as in many countries in Southeast Asia. Because of the anti-inflammatory and analgesic properties of SPL [18], *Spilanthes acmella* is also known as “Toothache Plant” or “Electric Daisy” in the U.S.A. Furthermore, while the antimicrobial activity of SPL was demonstrated against *Klebsiella pneumoniae* [20], *Trypanosoma brucei rhodesiense* and *Plasmodium falciparum* [21], the activity against Ct has not been investigated. 

First, we demonstrate that SPL suppresses Ct infection by increasing mitochondrial oxidation to a threshold that is detrimental to the Ct intracellular development, but not sufficiently high to impact the proliferation of non-infected host cells. Interestingly, upon infection of cells pre-treated with SPL, Ct suppresses the increase in mitochondria oxidation induced by SPL, taking control of host cell redox metabolism. Investigation of SPL mechanism of action raised the possibility that its activity is due to a chemical conversion to an endoperoxide derivative within the cellular milieu. This hypothesis was validated by the chemical synthesis of SPL endoperoxide (SPL^E^), which showed more potent activity against Ct infection. Studies with recombinant antioxidant proteins, revealed that the SPL^E^ but not SPL reacts preferentially with the mitochondrial protein peroxiredoxin 3 (Prx3), an activity likely to be responsible for the shift to a more oxidative state of host cell mitochondria. Overall, the studies presented here identify the reliance of Ct infection on mitochondrial redox state as the Achilles’ heel to be exploited for prevention or treatment of this disease, establish SPL or its SPL^E^ derivative as natural compounds with anti-chlamydial activity, and SPL^E^ as a scaffold for the development of selective inhibitors against mitochondrial Prx3. The data demonstrate for the first time the reaction of peroxiredoxins with endoperoxides and suggest a novel oxidizing activity distinguishing Prx2 and Prx3 from Prx1.

## 2. Materials and Methods 

### 2.1. Reagents

Antibodies were obtained from the following sources: goat anti-*C. trachomatis* MOMP (Meridian Life Sciences, Saco, ME, USA), rhodamine red X conjugated anti-goat secondary antibody (Jackson Laboratories, West Grove, PA, USA), HRP conjugated anti-rabbit secondary antibody (Cell Signaling Technologies, Danvers, MA, USA), rabbit anti-HSP60 (Santa Cruz Biotechnology, Santa Cruz, CA, USA), and HRP conjugated anti-mouse secondary antibody (Cell Signaling Technologies, Danvers, MA, USA). Fetal bovine serum (FBS) (Atlanta Biologicals, Flowery Branch, GA, USA), Dulbecco’s modified Eagle medium (DMEM) (Invitrogen, Grand Island, NY, USA) and phosphate buffered saline (PBS) were purchased from (Lonza, Walkersville, MD, USA). Ethyl acetate, ammonium formate, and dichloromethane were obtained from ACROS Organics (Pittsburgh, PA, USA) and 3,3′-diindolylmethane was from Santa Cruz Biotechnology, Santa Cruz, CA. Acetonitrile and water were HPLC grade from Fisher Scientific and Sep-Pak Vac 3cc (200mg) certified silica cartridges were purchased from Waters [22], part No: 186004614. Spilanthol was obtained from Santa Cruz Biotechnology (Dallas, TX, USA; sc-474036) and spilanthol endoperoxide (SPL^E^) was synthesized as described below. 

### 2.2. Chemical Synthesis of SPL^E^

Ethyl (E)-6-oxohex-2-enoate (**3**). To a solution of **2** [23] (1 g, 6.32 mmol) in CH_2_Cl_2_ (50 mL) was added pyridinium chlorochromate (PCC) (1.5 eq., 2.04 g, 9.48 mmol) and silica (2 g). The reaction stirred at 25 °C for 6 h and the solvent was removed *in vacuo*. The resulting brown powder was filtered through a short silica pad using 5:1 hexane:ethyl acetate to give **3** (0.75 g, 4.8 mmol, 76%), which was used without further purification. NMR data matched that reported in [24]. The NMR spectra of **3** and other chemical products are included in the Supporting Information. ^1^H NMR (400 MHz, Chloroform-*d*) δ 9.80 (t, *J* = 1.1 Hz, 1H), 6.94 (dt, *J* = 15.6, 6.6 Hz, 1H), 5.85 (dt, *J* = 15.7, 1.6 Hz, 1H), 4.18 (q, *J* = 7.1 Hz, 2H), 2.65 (tt, *J* = 7.0, 1.0 Hz, 2H), 2.58–2.48 (m, 2H), 1.32–1.25 (m, 4H).


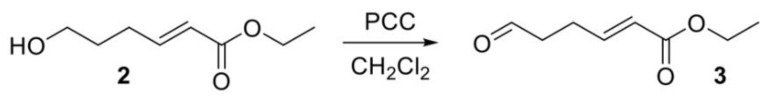


Ethyl (2E,6E/Z,8E/Z)-deca-2,6,8-trienoate (**5**). To a suspension of **4** (1.6 eq., 0.99 g, 2.81 mmol) in 50 mL of diethyl ether was slowly added n-butyllithium (n-BuLi) (1.5 eq., 1.05 mL, 2.5 M in hexane) at −78 °C. The bath was removed, and the mixture stirred for 30 min. Then the mixture was cooled again to −78 °C, **3** (1 eq., 0.27 g, 1.76 mmol) was added, and the reaction mixture was stirred for 1h at −78°C and the allowed to warm to ambient temperature overnight. Acetone was added to quench excess reagent and the mixture was filtered through Celite. Solvent was removed *in vacuo* to give an oily residue. The residue was partitioned between ethyl acetate and brine. The organic layer was dried over MgSO_4_ and evaporated. The crude product was purified by chromatography over silica (20:1 hexane/ethyl acetate) to yield **5** as a mixture of diastereomers (0.3 g, 1.57 mmol, 89%). NMR data matched that for the two isomers reported in [25]. ^1^H NMR (400 MHz, Chloroform-*d*) mixture of diasteromers δ 6.96 (dq, *J* = 15.7, 6.6 Hz, 1H), 6.45–5.12 (m, 5H), 4.18 (qd, *J* = 7.1, 0.9 Hz, 2H), 2.42–2.16 (m, 4H), 1.82–1.71 (m, 3H), 1.31–1.26 (m, 3H).


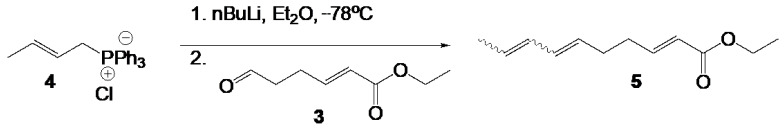


(2E,6E/Z,8E/Z)-deca-2,6,8-trienoic acid (**6**). To a THF (15 mL) and water (5 mL) solution of **5** (0.2 g, 1 eq., 1.06 mmol) was added LiOH monohydrate (5 eq., 0.22 g, 5.29 mmol). The mixture was stirred at reflux for 14 h. The solution was cooled to ambient and THF removed *in vacuo*. Aqueous HCl was added to the mixture until pH = 2. The cloudy mixture was extracted with ethyl acetate 3 × 50 mL. The combined organic layers were washed with brine and dried over MgSO_4_. Removal of solvent *in vacuo* gave **6** as a mixture of diastereomers (0.174 g, 1.05 mmol, 99%). NMR data matched that in [26]. ^1^H NMR (400 MHz, Chloroform-*d*) mixture of diastereomers δ 11.56 (s, 1H), 7.15–7.01 (m, 1H), 6.42–6.18 (m, 1H), 6.09–5.91 (m, 1H), 5.84 (ddt, *J* = 15.6, 6.1, 1.5 Hz, 1H), 5.76–5.37 (m, 2H), 2.46–2.13 (m, 4H), 1.86–1.63 (m, 3H). ^13^C NMR (101 MHz, CDCl_3_) mixture of diastereomers δ 178.46, 172.22, 171.35, 151.38, 151.34, 151.29, 151.26, 131.79, 131.54, 131.29, 131.13, 130.22, 129.82, 129.41, 129.14, 129.09, 128.33, 127.87, 127.11, 127.01, 126.58, 125.99, 125.00, 124.60, 124.08, 121.18, 121.15, 121.12, 120.70, 77.39, 77.07, 76.76, 60.48, 32.68, 32.37, 32.24, 32.15, 31.94, 31.11, 30.80, 30.40, 29.72, 29.38, 26.13, 25.98, 25.80, 22.71, 21.02, 18.30, 18.01, 14.18, 14.12, 13.30, 13.17.


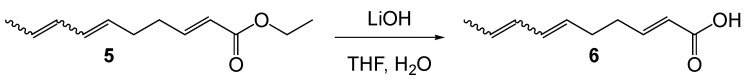


Spilanthol (SPL). A mixture of **6** (1 eq., 0.22 g, 1.30 mmol) and *N*-Ethyl-*N*′-(3-dimethylaminopropyl)carbodiimide (EDC) (1.35 eq., 0.27 g, 1.76 mmol) was dissolved in CH_2_Cl_2_ and cooled to 0 °C. Isobutylamine, **7**, (1 eq., 71.5 mg, 1.30 mmol) and 4-dimethylaminopyridine (DMAP) (0.1 eq., 15.9 mg, 0.13 mmol) was added. The mixture was stirred for 2h at 0 °C and then allowed to warm with the bath to ambient temperature overnight. The reaction mixture was diluted with brine and the layers separated. The aqueous layer was extracted 2 × 30 mL. The organic layers were combined and dried over MgSO_4_. Solvent was removed *in vacuo* and the product purified by chromatography over silica (2:1 hexane/ethyl acetate) to give SPL as a mixture of diastereomers (0.158g, 0.72 mmol, 55%; 45% (2E, 6E, 8E), 40% (2E, 6Z, 8E), 15% other). ^1^H NMR (400 MHz, Chloroform-*d*) δ 6.82 (dt, *J* = 15.1, 6.7, 1H), 6.38–6.17 (m, 1H), 6.07–5.91 (m, 1H), 5.86–5.36 (m, 3H), 5.26 (dt, *J* = 10.8, 7.2 Hz, 1H), 3.21–2.80 (m, 2H), 2.36–2.16 (m, 3H), 1.86–1.66 (m, 4H), 0.96–0.88 (m, 6H).


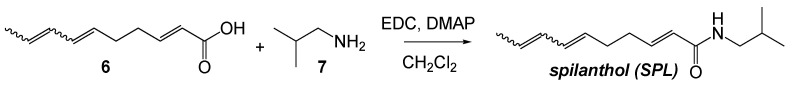


Singlet oxygenation of SPL. To a solution of SPL (1 eq., 20 mg, 0.103 mmol) in CH_2_Cl_2_ (25 mL) was added Rose Bengal (5 mol%). The solution was stirred at 0–5 °C under white light with a constant stream of dry air bubbled at the base of the flask for 6h. After TLC indicated consumption of starting material, the reaction was filtered and concentrated *in vacuo*. The residue was purified by preparative thin layer chromatography (TLC) over silica (10% MeOH in CH_2_Cl_2_ with 1% Et_3_N) to yield *rac*-**SPL^E^** (11.4 mg, 0.045 mmol, 44%; Exact mass 253.168; Calculated mass + H^+^ 254.176; Mass measured using electrospray ionization time-of-flight mass spectrometry: 254.167). NMR data is consistent with that reported in [21]. ^1^H NMR (400 MHz, Chloroform-*d*) δ 6.82 (dt, *J* = 15.1, 7.0 Hz, 1H), 5.93 – 5.73 (m, 3H), 5.48 (s, 1H), 4.78–4.63 (m, 1H), 4.40 (dd, *J* = 8.9, 2.6 Hz, 1H), 3.22–3.11 (m, 2H), 2.50–2.24 (m, 2H), 1.95–1.64 (m, 4H), 1.25 (d, *J* = 6.7 Hz, 4H), 0.92 (d, *J* = 6.7 Hz, 6H. ^13^C NMR (101 MHz, CDCl_3_) δ 165.88, 143.41, 129.42, 127.04, 124.47, 77.24, 74.38, 46.86, 31.61, 28.59, 27.94, 20.12, 18.13.


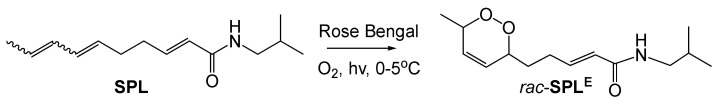


### 2.3. Cell Culture

HeLa cells were purchased from ATCC, USA and cultured in antibiotic-free DMEM containing 10% FBS at 37 °C and 5% CO_2_. 

### 2.4. Formulation of Chlamydia Trachomatis Elementary Body (EB) Stock Solution 

HeLa cells were grown to 80% confluency followed by infection with Ct serovars A or D in the presence of 1 μg/mL cycloheximide (Sigma-Aldrich, St. Louis, MO, USA) and 6% glucose (sterile) for 48 h. Following infection, cells were lysed by centrifuging with autoclaved glass beads for ten minutes at 2,000 rpm at 4 °C. The supernatant was collected and subjected to further centrifuging for one hour at 16,000 rpm at 4 °C. The resulting supernatant was discarded. The remaining bacterial pellet was resuspended in ice-cold SPG, a crystallization stock solution made up of succinic acid (S), sodium dihydrogen phosphate (P), and glycine (G). Suspended EBs were aliquoted and stored at −80 °C until use. 

### 2.5. Chlamydia Trachomatis Infection 

HeLa cells were infected with Ct EBs serovars A or D using MOI 2 in the presence of 6% glucose in antibiotic-free DMEM. Cells were further treated as described below for each individual condition and then processed for analysis. Vehicle control for Ct infection was either SPG or heat-inactivated Ct EBs (56 °C, 30 min). 

### 2.6. SPL, SPL^E^, and MitoPQ (MitoParaquat) Treatment of Infected Cells

HeLa cells were incubated with SPL or SPL^E^ at the concentrations indicated in the respective figure legends at the time of Ct infection or at 24 h post infection (hpi) at a MOI of 2. Treatment with vehicle solution (e.g., 0.1% DMSO) was used as control. Treatment with MitoPQ (20 μM) [14] or narciclasine (5 nM) [27] was used to validate the mitochondrial mechanisms. 

### 2.7. Cell Proliferation Assay

Cell proliferation was calculated by imaging the cells over time using Essen Bioscience IncuCyte ZOOM live-cell imager and extracting the % confluence from imaging data. 

### 2.8. Confocal Imaging

HeLa cells were seeded on 8-well EZ glass slides (Millipore, Temecula, CA, USA) in antibiotic-free DMEM. After Ct infection and treatment, the cells were washed with PBS and fixed in 4% paraformaldehyde for 15 min, washed three times with PBS and permeabilized with 0.1% TritonX-100 for 10 min at 25 °C. Cells were further washed with PBS and blocked with 1% BSA for one hour at 25 °C. Cells were incubated with goat anti-Ct EB (Meridian Life Sciences, Saco, ME, USA) targeting the major outer membrane protein (MOMP) overnight at 4 °C, followed by addition of the rhodamine-conjugated anti-goat secondary antibody (Jackson Laboratories, West Grove, PA, USA) for one hour at 25 °C. F-actin was detected with Alexa Fluor 488-phalloidin (green). The slides were then mounted with Vecta shield mounting medium containing DAPI for fluorescence staining of host cell nuclei (Vector Laboratories, Burlingame, CA, USA) and #1.5 glass coverslips. Confocal imaging was performed using a Zeiss LSM880 with a 40×/0.95 objective at 405 nm (DCP-NEt_2_C, [28]), 488 nm (F-actin), and 561 nm (Ct inclusions) wavelengths. The percent area occupied by Ct inclusions was quantified with ImageJ [29] and data were fitted to calculate EC50. 

### 2.9. Analysis of Mitochondrial Protein Sulfenylation by Flow Cytometry 

HeLa cells were seeded on 6-well culture plates and allowed to reach 80% confluency. Cells were then treated with SPL alone (120 μM) or SPL and Ct (SPL 120 μM, Ct MOI of 2). At 24 hpi, the cells were incubated with 50 μM DCP-NEt_2_C for thirty-minute at 37 °C in complete media to label mitochondrial sulfenylated proteins [28]. Cells were then trypsinized, washed with ice-cold PBS, and fixed in 100% methanol for ten minutes on ice. The cells were washed then three times with FACS wash buffer and finally resuspended in 300 µL FACS buffer for analysis. Flow cytometry analysis was performed at 405 nm wavelength using a BD FACSCanto II flow cytometer. 

### 2.10. Transmission Electron Microscopy Imaging

HeLa cells were treated with Ct alone (MOI 2) and Ct in combination with SPL (120 μM). At 24 hpi, the cells were washed with PBS and fixed with 2.5% glutaraldehyde in 0.1 M Millonig’s buffer (pH 7.3) for one hour followed by addition of 1% osmium tetroxide in 0.1 M Millonig’s buffer for thirty minutes. The cells were washed three times with 0.1 M Millonig’s buffer and dehydrated with 50% and 100% ethanol. The cells were then scraped and centrifuged to obtain a pellet. The pellets were processed in propylene oxide two times fifteen minutes each and further infiltrated with 1:1 solution of Spurr’s resin and propylene oxide for one hour, followed by a 2:1 solution of resin and propylene oxide for two hours, and finally into a pure solution of resin for one hour. The resin infiltrated pellets were kept overnight in a 70 °C oven and the pellets were sectioned into 90 nm thin sections using a Reichart-Jung ultramicrotome. Sections were stained with uranyl acetate and lead citrate and viewed under FEI Tecnai BioTwin electron microscope (HV = 80.0 kV; direct magnification 9300×). 

### 2.11. Recombinant Proteins and Electrospray Ionization Time-of-Flight Mass Spectrometry (ESI-TOF MS)

Human wild-type recombinant Prx1, Prx2 and Prx3, His-tag Prx3, and sulfiredoxin (Srx) were expressed in *Escherichia coli* and purified essentially as described earlier [30,31,32]. The proteins (100 μM) were reduced with 10 mM DTT in 50 mM ammonium bicarbonate pH 8.0 for one hour at room temperature. Reduced Prx was then passed through a Bio-Spin Bio-Gel P-6 equilibrated with 50 mM ammonium bicarbonate pH 8.0 to remove DTT. An aliquot of the resulting protein was saved as control, and two other aliquots were incubated at room temperature for one hour with 1 mM of either SPL or SPL^E^. The reaction mixture was further passed through a Bio-Spin Bio-Gel P-6 column equilibrated with 50 mM ammonium bicarbonate pH 8.0, and the protein was analyzed along with the reduced protein by ESI-TOF MS. ESI-TOF MS analysis was performed on an Agilent 6120 MSD-TOF system operating in positive ion mode with the following settings: capillary voltage of 3.5 kV, nebulizer gas pressure of 30 psig, drying gas flow of 5 L/min, fragmentor voltage of 175 V, skimmer voltage of 65 V, and dry gas temperature of 325 °C. Samples were introduced via direct infusion at a flow rate of 20 μL/min using a syringe pump (KD Scientific). Mass spectra were acquired over the range of 500-3200 m/z, then averaged, deconvoluted, and ion abundance quantified using Agilent MassHunter Workstation software v B.02.00. 

### 2.12. Data Analysis

Statistical analyses for cell proliferation were performed using Microsoft Excel and all results are displayed as mean ± SEM. The significance of the difference of means was determined by student’s *t*-test and p values of <0.05 were considered significant. P values were further confirmed using the GraphPad QuickCalcs. Flow cytometry data were analyzed as described previously [28,33]. Briefly, effect size was calculated by finding the difference in the means between the control and treatment conditions and dividing by the larger SD of the two groups. Effect sizes were classified as very small (x < 0.2), small (0.2 < x < 0.4), medium (*) (0.4 < x < 0.7), large (**) (0.7 < x < 1.2), and very large (***) (x > 1.3). Data analysis for EC50 calculation was performed in GraphPad and data are shown as ±SEM based on three biological replicates. 

## 3. Results

### 3.1. SPL Inhibits Intracellular Ct Development 

**Figure 1:**
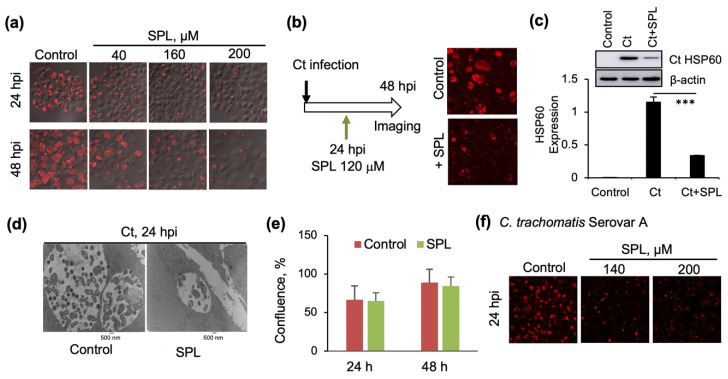
Inhibition of Ct infections by spilanthol (SPL): (**a**) Confocal microscopy imaging of cells infected with *Chlamydia trachomatis* (Ct) serovar D and SPL vehicle control (DMSO) at 24 h (upper) or 48 h post infection (hpi) (lower) or with SPL followed immediately by infection with Ct serovar D, and similarly imaged at 24 or 48 hpi. Chlamydial inclusions were visualized with anti-chlamydial major outer membrane protein (MOMP) antibody (red) and the image was overlayed with phase contrast image of cells. (**b**) Similar to panel (**a**) with the difference that SPL (120 μM) was added at 24 hpi and then the cells were imaged after additional incubation for 24 h (48 hpi). (**c**) Quantification of the effects of SPL on Ct infection by Western blot analysis of chlamydial HSP60 protein (*** *p <* 0.001). Control represents cells treated with heat-inactivated Ct and 0.1% DMSO. (**d**) Transmission electron microscopy (TEM) imaging (9,300×) comparing Ct inclusions of SPL (120 μM) treated and vehicle (DMSO) treated cells at 24 hpi. The small darker features within the inclusion are the elementary bodies (EBs) while the larger lighter gray features are the reticulate bodies (RBs). (**e**) The effects of SPL (120 μM) on the proliferation of HeLa cells in the absence of Ct infection were quantified by analyzing the percent confluence over time using the IncuCyte ZOOM live-cell imager. The data at 24 and 48 h were extracted and plotted as bar graph. There was no statistically significant difference between control and SPL treatment conditions at either one of the timepoints. (**f**) As in panel (**a**), with the difference of infection being performed with Ct serovar A. All data representative of a minimum two biological replicates.

Given our earlier findings of sulforaphane promoting the intracellular development of Ct [14], we started our investigations by testing the pro- or anti-chlamydial (Ct serovar D) activity of SPL in HeLa cells, a widely accepted and validated in vitro model for mechanistic studies of sexually transmitted infections. The effects of SPL were monitored by confocal imaging of intracellular inclusions, the protective vacuoles within which Ct amplifies. The results revealed inhibition of Ct infection by SPL regardless of whether the compound was added to HeLa cells before Ct infection (Figure 1a) or at 24 h post-infection (Figure 1b). The confocal imaging results were further confirmed by monitoring chlamydial heat shock protein 60 (HSP60), an indicator of chlamydial load (Figure 1c), and transmission electron microscopy showing smaller inclusions with a lower number of EBs and RBs (Figure 1d). Importantly, SPL did not impact host cell proliferation demonstrating it targets Ct-induced events (Figure 1e). The activity of SPL was then further tested using infection of HeLa cells with Ct serovar A, responsible for ocular infections and trachoma (Figure 1f). The results showed comparable anti-chlamydial effects, demonstrating that the activity of SPL was not dependent on a specific Ct serovar. These initial results were very promising as they revealed SPL’s potential utilization for both the prevention or treatment of Ct infections. All subsequent studies described in the next sections were performed with Ct serovar D and HeLa cells.

### 3.2. SPL Increases Mitochondrial Oxidative State and Disrupts the F-Actin Ring Supporting the Ct Inclusion

**Figure 2:**
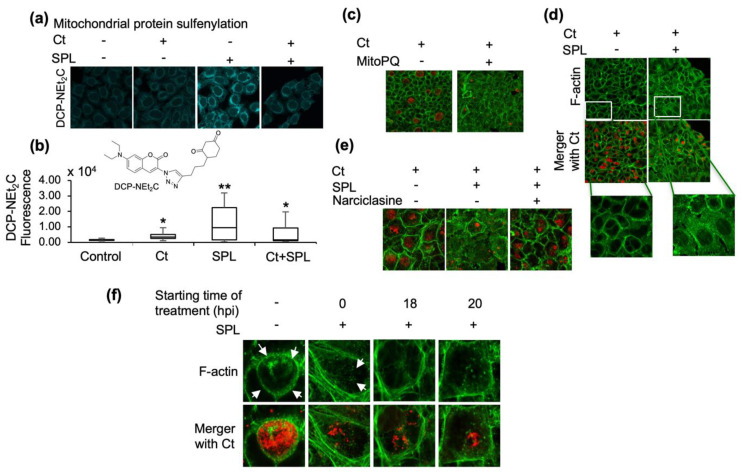
SPL disrupts the formation of F-actin ring and stability of Ct inclusions by increasing mitochondrial oxidative state. (**a**) Confocal imaging analysis of mitochondrial protein sulfenylation induced by Ct and SPL (120 μM) alone or when used in combination. Mitochondrial sulfenylated proteins were imaged with DCP-NEt_2_C; (**b**) Flow cytometry analysis of DCP-NEt_2_C labeled cells to further validate and quantify the effects of Ct and/or SPL treatments. Statistical significance is indicated by the effects size as medium (*, 0.4 < x < 0.7) or large (**, 0.7 < x < 1.2) (*n* = 2); (**c**) Disruption of F-actin cytoskeletal organization and Ct inclusions by MitoPQ, a mitochondria-targeted ROS generator. F-actin was detected with Alexa Fluor 488-phalloidin (green) and chlamydial inclusions were detected using anti-MOMP antibody (red); (**d**–**f**) The same imaging analysis monitoring Ct inclusions and F-actin was performed to investigate the effect of SPL on Ct inclusions and F-actin network (**d**), the rescue of SPL detrimental effects by narciclasine (**e**), and to demonstrate that SPL disrupts the F-actin organization and the ring structure surrounding the Ct inclusion regardless of the time of addition to cells relative to Ct infection (control, Ct infection 24 hpi, 0.1% DMSO). All imaging data are representative of a minimum two biological replicates and were collected at 40× magnification. White arrows point to the F-actin ring.

Considering the critical function of mitochondria in the regulation of cellular redox processes, we have focused our subsequent studies on the role of mitochondria redox state in Ct infection and the impact of SPL on mitochondrial protein oxidation. For these studies we have used selective labeling of protein sulfenylation at 24hpi with fluorescent mitochondria-targeted DCP-NEt_2_C probe [28] and performed confocal imaging (Figure 2a) and flow cytometry analysis (Figure 2b) of cells treated with Ct and SPL alone or in combination. While Ct induced a statistically significant but low increase in mitochondrial protein oxidation, a much higher protein oxidation level was noted in cells treated with SPL (Figure 2a,b). Interestingly, Ct was able to significantly suppress the increase in mitochondrial protein sulfenylation induced by SPL, to a level closer to that observed with Ct treatment in the absence of SPL (Figure 2a,b). In a previous publication, we utilized MitoPQ, a generator of mitochondrial ROS to increase protein sulfenylation in HeLa cells [14] and showed this to be detrimental to Ct infection. Here we sought to further investigate if the increase in mitochondrial ROS and protein sulfenylation by MitoPQ or SPL could disrupt the F-actin cytoskeletal organization critical to the formation of the F-actin ring sustaining the Ct inclusions. First, HeLa cells were treated with MitoPQ in the absence of SPL, and we monitored both the inclusions (red, Figure 2c) and the host cell F-actin (green, Figure 2c). Consistent with previously reported data [14], the results show inhibition of Ct infection, suggesting that the main mechanism of Ct inhibition by SPL is through disruption of mitochondrial redox metabolism. Also notable in the MitoPQ data was the loss of cytoskeletal F-actin organization (Figure 2c). To investigate if this is also a feature of SPL treatment, the same imaging analysis was performed in cells infected with Ct, with and without SPL treatment (Figure 2d), and using narciclasine to rescue mitochondrial metabolism [27] (Figure 2e). Indeed, the results show a general disruption of F-actin organization by SPL and prevention of this activity along with the rescue of Ct intracellular development by narciclasine. Further confocal imaging analysis focusing on the F-actin ring surrounding the intracellular inclusion demonstrates the damaging effects of SPL on this structure when added either at the time of infection or after infection (Figure 2f). 

### 3.3. The Endoperoxide Derivative of SPL (SPL^E^) Is a More Potent Inhibitor of Ct Infections and Similarly Interferes with the Organization of the F-Actin Ring 

**Figure 3:**
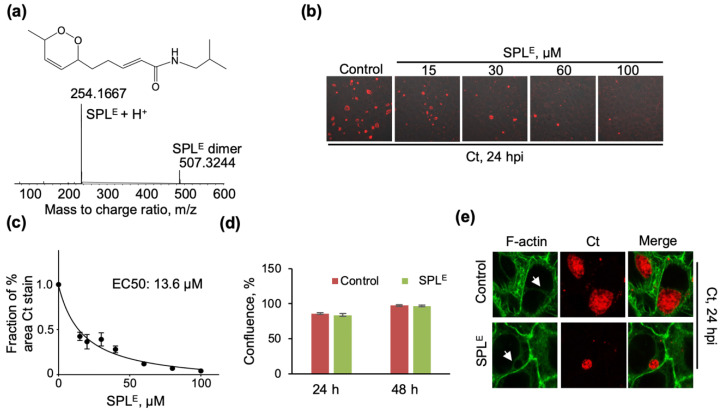
SPL^E^ shows more potent inhibition of Ct infection (**a**) Chemical structure and ESI-TOF MS data of SPL^E^ synthesized as described in Materials and Methods. MS data also shows the presence of SPL^E^ dimer formed under the ESI-TOF MS conditions; (**b**) Confocal microscopy imaging of cells infected with Ct and SPL^E^ vehicle (0.1% DMSO) at 24 hpi (control) or with SPL^E^ followed immediately by infection with Ct and similarly imaged at 24 hpi. Chlamydial inclusions were visualized with anti-chlamydial major outer membrane protein (MOMP) antibody (red) and the image was overlayed with phase contrast images of cells; (**c**) Inhibition of Ct infection by SPL^E^ was quantified by calculating the % area stained with anti-MOMP and representing the data as a fraction of vehicle control (n = 3 biological replicates, 3–6 imaging areas per biological replicate); (**d**) The effects of SPL^E^ (120 μM) on the proliferation of HeLa cells in the absence of Ct infection were quantified by analyzing the percent confluence over time using the IncuCyte ZOOM live-cell imager. The data at 24 and 48 h were extracted and plotted as bar graph. There was no statistically significant difference between control and SPL treatment conditions at either one of the time points (n = 3). (**e**) Disruption of F-actin cytoskeletal organization and Ct inclusions by SPL^E^ (120 μM). F-actin was detected with Alexa Fluor 488-phalloidin (green) and chlamydial inclusions were detected using anti-MOMP antibody (red). All imaging data are representative of a minimum two biological replicates and were collected at 40x magnification. White arrows point to the F-actin ring.

Given the findings of increased mitochondrial oxidation state with SPL, we proceeded to investigate potential mechanisms leading to these effects. A starting hypothesis consistent with these observations was the possibility of SPL conversion to an endoperoxide derivative SPL^E^ in the mammalian cellular milieu by trapping singlet oxygen (^1^O_2_) species similar to what has been proposed in Acmella Ciliata [21] (referred to as dioxacmellamide in [21]). First, we proceeded with the chemical synthesis of SPL^E^ as described in Materials and Methods. This new route to SPL was designed to maximize the ability to prepare derivatives with structural variation in the diene tail and carboxyl substituent in the future. SPL was obtained as a mixture of geometric isomers of which approximately 40% was 2E, 6Z, 8E, the predominant isomer in commercial source of SPL used in this study. The mixture of SPL isomers was then converted to SPL^E^ by singlet oxygenation using Rose Begal as photosensitizer. Purification by TLC gave *rac-*SPL^E^, a racemic mixture of the 6R,9S- and 6S,9R-cis enantiomers (a single diastereomer). NMR and mass spectrometry data (Figure 3a; 0.009 delta mass between observed and calculated values) were consistent with this assignment (NMR data described in Materials and Methods and spectra shown in Supporting Information). Next, we tested the activity against Ct infection using the same set of assays as presented for SPL. SPL^E^ was more potent than SPL at inhibiting Ct infection (Figure 3b) with an EC50 of 13.6 μM (Figure 3c) without impacting cellular proliferation (Figure 3d). The effects of SPL^E^ on the F-actin ring were similar to those of SPL (Figure 3e).

### 3.4. SPL^E^ Reacts Predominantly with Mitochondrial Antioxidant Protein Peroxiredoxin (Prx3) 

**Figure 4:**
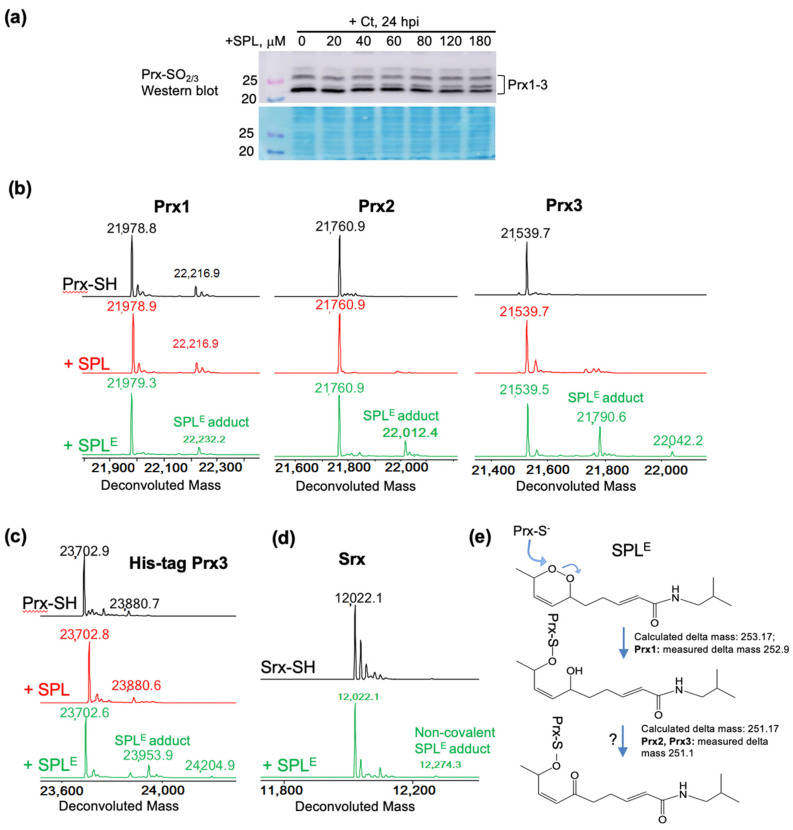
Reaction of SPL^E^ but not SPL with reduced peroxiredoxins. (**a**) Western blot analysis of hyperoxidized Prx(1–4) in HeLa cells showing that SPL does not increase the level of hyperoxidized Prx in Ct infected cells. Protein stain is shown to illustrate equal loading of cell lysates; (**b**) ESI-TOF MS data of Prx1, Prx2 and Prx3 after reduction with DTT (black upper spectra), and after the reaction with SPL (red middle spectra) or SPL^E^ (green lower spectra). The results show lack of reaction with SPL and preferred reaction of SPL^E^ with Prx3, as well as formation of a more oxidized adduct with Prx2 and Prx3 (delta mass +251.1) compared with Prx1 (delta mass +252.9); (**c**,**d**) The same reaction as in (**b**) but using a His-tagged construct of Prx3 (**c**), showing the dependence of the reaction on the oligomeric structure of native Prx3, and using Srx (**d**) showing lack of reaction with this protein; (**e**) Potential mechanisms for the reaction of SPL^E^ with reduced Prx1, Prx2, and Prx3.

Peroxiredoxins are key intracellular enzymes involved in detoxification of oxidants (e.g., H_2_O_2_) and oxidized species (e.g., lipid peroxides), connecting phosphorylation and redox signaling [34]. These enzymes can be inactivated at high levels of H_2_O_2_ or lipid peroxides during turnover leading to the formation of sulfinic acid at the catalytic peroxidatic cysteine (hyperoxidation) or by posttranslational modifications. The hyperoxidized inactive species can be repaired by sulfiredoxin (Srx), restoring their antioxidant activity. Amongst the 6 mammalian isoforms, Prx3 is located in the mitochondria and is the key regulator of mitochondrial H_2_O_2_. Prx1 and Prx2 belong to the same family of 2-Cys peroxiredoxins as Prx3, but are predominantly located in the cytosol. An increase in overall mitochondrial protein sulfenylation (Figure 2a,b) with SPL treatment could ensue if Prx3 is inactivated during SPL/SPL^E^ treatment by either hyperoxidation at the catalytic peroxidatic cysteine residue or by covalent adduct formation with SPL or SPL^E^. Covalent adduct formation of Prx3 with SPL or SPL^E^ was deemed feasible considering the nucleophilic properties of the peroxidatic cysteine residue (Cys108 in Prx3) and the presence of α,β-unsaturated carbonyl in SPL and SPL^E^ amenable to Michael addition, and the reactive endoperoxide in SPL^E^. Western blot analysis did not indicate substantial changes in Prx3 hyperoxidation during the time course of SPL treatment of Ct infected cells (Figure 4a). To investigate the possibility of SPL or SPL^E^ reaction with Prx3 and adduct formation resulting in inhibition of Prx3 activity, we performed side-by-side mass spectrometry analysis of recombinant Prx1, Prx2, and Prx3 reaction with SPL and SPL^E^ (Figure 4b). Interestingly, SPL^E^ but not SPL reacted with mitochondrial Prx3, providing an explanation for increased mitochondrial protein sulfenylation and anti-chlamydial activity of SPL^E^. Further experiments were conducted with a His-tagged version of Prx3, which previous studies using size exclusion chromatography multiple angle light scattering (SEC-MALS) showed to reside in concatenated (two intertwined) dodecamers compared to the dodecameric wild-type reduced Prx3 [35]. Although there was an adduct observed with SPL^E^, this was of lower abundance compared with wild-type Prx3 (Figure 4c). Lastly, we investigated the reaction of SPL^E^ with sulfiredoxin (Srx), both because of the Srx function in the repair of hyperoxidized inactive 2-Cys peroxiredoxins, and also as another protein containing a reactive cysteine to gauge the relative selectivity of SPL^E^ toward Prx3. SPL^E^ did not react with Srx, the trace amount of adduct observed corresponded to a non-covalently bound species (Figure 4d). Based on the average delta mass for the covalent adduct of Prx3 with SPL^E^ (+251.1 Da) and the lack of reaction with SPL, we propose a reaction mechanism by which the peroxidatic cysteine in Prx3 attacks the endoperoxide, forming a sulfenate adduct (sulfenate ester), which seems to be further oxidized by an unknown mechanism. Interestingly, although the Prx2 adduct also corresponded to a mass shift of +251.1 Da, for Prx1 this was +252.9 (~2 protons). Based on these observations we envisioned a chemical path (Figure 4e), which we further rationalize in the Discussion. 

## 4. Discussion

Regulation of redox metabolism is one of the cell’s primary defense response to bacterial infection [36,37]. Indeed, Ct infections have been reported to induce an increase in intracellular reactive oxygen species (ROS) [38,39,40], in part mediated by transient activation of NADPH oxidase (NOX) [39,41]. By examining disulfide bond formation and cross-linking in the chlamydial outer membrane, it has been determined that Ct exists in a more reduced environment during early infection, gradually shifting to a more oxidized but non-apoptotic state in later stages of the developmental cycle [42]. Interestingly, there are also reports of chlamydial infections actually protecting cells from H_2_O_2_-induced apoptosis [43]. 

These findings have led to the exploration of compounds with redox-altering properties for prevention and/or treatment of chlamydial infections (e.g., ascorbic acid [38]). Our efforts focused on examining the potential anti-chlamydial activity of natural compounds with reported anti-inflammatory and antioxidant activities. Surprisingly, the first compound we investigated, sulforaphane—a key compound in broccoli and other cruciferous vegetables, promoted chlamydial infection by suppressing mitochondrial protein sulfenylation and inducing activation of complement C3 in mammalian host cells [14]. Given these intriguing findings, we proceeded to test the anti/pro-chlamydial activity of other natural plant products with reported anti-inflammatory activity. We selected to work with SPL (described in the Introduction) because of its established antimicrobial activity against several intracellular pathogens, unexplored activity against Ct, and its anti-inflammatory and redox-regulatory properties. 

The very first studies (Figure 1) revealed a diametrical opposite anti-chlamydial effect of SPL compared to the infection-promoting activity of sulforaphane [14]. Importantly, the studies showed that this activity was not serovar-dependent being effective against both Ct serovars A and D, and not dependent on the time of treatment suppressing Ct infections when added either at the time of treatment or after the establishment of intracellular inclusions. The data also showed that these effects are not due to the killing of host cells as SPL treatment did not alter cell growth. 

As sulforaphane activity was linked in our previous studies to a decrease in mitochondrial protein sulfenylation, the next experiments were directed to quantify the effects of SPL on these species (Figure 2a,b). Indeed, confocal imaging using a mitochondria-targeted chemical probe selective for protein sulfenylation showed significantly increased oxidation with SPL treatment, which then Ct was able to suppress but down to levels that were still higher than those induced by Ct alone. This analysis reveals the very precise regulation of host cell mitochondrial redox metabolism by Ct to a level that is just sufficiently high to support its intracellular development and not detrimental to host cell. The data also shows that Ct can regulate mitochondrial protein sulfenylation both up from basal levels in host cells and down from the SPL-induced levels upon infection. The critical function of mitochondrial metabolism and redox state in Ct infection was further validated with the MitoPQ and narciclasine studies. The induction of mitochondrial ROS and protein sulfenylation by MitoPQ proved detrimental to Ct infection consistent with our earlier findings [14]. Here we show that MitoPQ also induced a loss of F-actin cytoskeletal network and prevented the organization of the F-actin ring around the chlamydial inclusion (Figure 2c), similar to the effects of SPL (Figure 2d). Narciclasine, a natural compound with mitochondrial ROS suppressing activity, rescued the SPL-induced damage to F-actin organization and Ct infection (Figure 2e). Importantly, when SPL was added after establishment of chlamydial inclusions (e.g., at 18 and 20 hpi), it was still able to disrupt the inclusion and inhibit intracellular Ct expansion. 

The increase in mitochondrial protein sulfenylation with SPL was puzzling as SPL and the plant extracts from which it originates are generally considered as antioxidants [44]. A hypothesis to resolve this apparent contradiction was that within the cellular environment SPL may convert to an endoperoxide derivative (SPL^E^) by trapping ^1^O_2_, which would be responsible for its perceived antioxidant activity, and then the SPL^E^ (an oxidant) would induce the increase in protein sulfenylation. The first part of this hypothesis is supported by the identification of SPL^E^ in plant extracts from *Acmella ciliata* [21], and the chemical synthesis of SPL^E^ from SPL by in situ generation of ^1^O_2_ described in Materials and Methods. Indeed, SPL^E^ showed stronger activity against Ct infection resulting in disruption of F-actin cytoskeletal organization similar to the effects of SPL (Figure 3).

Finally, we wanted to go one step further and investigate potential targets of SPL or SPL^E^ that would lead to increased mitochondrial protein sulfenylation. The main mitochondrial antioxidant enzyme responsible for the suppression of H_2_O_2_, a reactive oxygen species leading to protein sulfenylation, is peroxiredoxin isoform 3 (Prx3). This enzyme belongs to the 2-Cys class of peroxiredoxins cycling between the reduced, sulfenylated upon reaction with H_2_O_2_ or lipid peroxides, and intermolecular disulfide states during the catalytic cycle. The disulfide oxidized protein is reduced back to the active state by the mitochondrial thioredoxin 2/thioredoxin reductase 2/NADPH system. Amongst mammalian Prx isoforms, Prx3 is the most resistant to inactivation by hyperoxidation (Prx-C_P_-SO_2_H; C_P_ denotes the peroxidatic/reactive cysteine), which can nevertheless occur at high levels of peroxide (H_2_O_2_ or lipid peroxides) [30,31,45]. The possibility of repair exists, and it is catalyzed by sulfiredoxin allowing Prx to re-enter the catalytic cycle. Interestingly, side-by-side monitoring of SPL or SPL^E^ adduct formation with recombinant Prx3 and the cytosolic Prx1 and Prx2, revealed that SPL did not react at any significant extent with any of the Prx isoforms, and SPL^E^ reacted preferentially with Prx3. This profile is consistent with the hypothesis of SPL to SPL^E^ conversion within the cell or perhaps more selectively within the mitochondria, and partial inhibition of Prx3 activity leading to the observed increase in host cell mitochondrial sulfenylation. To our knowledge, this is the first report of a peroxiredoxin reaction with endoperoxides. However, the chemical nature of the adducts and the reaction path to these remain to be elucidated. Given the lack of reaction of the peroxiredoxins with SPL, the Michael addition at the α,β-unsaturated carbonyl is unlikely although possibly the binding and/or reaction of the endoperoxide group could position the unsaturated carbonyl for reaction with a protein nucleophile. Assuming the reaction occurs similar to the reaction with H_2_O_2_ (H-O-O-H) or lipid peroxides (R-O-O-H), the peroxidatic cysteine could attack at either one of the two oxygens in the R-O-O-R sequence of the endoperoxide leading to a sulfenate ester and a hydroxyl (C_P_-S-O-R~R-OH). This is close in mass to the adduct observed in Prx1. However, the adduct observed in Prx2 and Prx3 is ~2 Da smaller than this suggesting a potential subsequent oxidation event or a different chemical path. We considered the attack of C_P_-S-O-R~R-OH by a nucleophile at the α,β-unsaturated carbonyl within the Prx monomer, but this reaction does not result in additional loss of 2 Da. This very intriguing observation remains to be explored in future studies. 

## 5. Conclusions

A large drawback for treatment of Ct infections is the absence of a vaccine and of low-cost drugs, as many patients particularly in third world countries cannot afford the cost of antibiotic treatment. In addition, there are also other problems with antibiotic treatment in developing nations, including the prevalence of expired antibiotics being donated to these countries [46,47,48], unhygienic conditions arising from lack of infrastructure and overpopulation [49,50,51], and the increasing concerns with antibiotic resistance. Overall, the studies presented here identify the reliance of Ct infection on mitochondrial redox state as the Achilles’ heel to be exploited for prevention or treatment of this disease, establish SPL or its SPL^E^ derivative as natural compounds with anti-chlamydial activity, and SPL^E^ as scaffold for development of selective inhibitors against mitochondrial Prx3.

## References

[1] Newman L., Rowley J., Vander Hoorn S., Wijesooriya N.S., Unemo M., Low N., Stevens G., Gottlieb S., Kiarie J., Temmerman M. (2015). Global Estimates of the Prevalence and Incidence of Four Curable Sexually Transmitted Infections in 2012 Based on Systematic Review and Global Reporting. PLoS ONE.

[2] Malhotra M., Sood S., Mukherjee A., Muralidhar S., Bala M. (2013). Genital Chlamydia trachomatis: An update. Indian J. Med. Res..

[3] Buckner L.R., Amedee A.M., Albritton H.L., Kozlowski P.A., Lacour N., McGowin C.L., Schust D.J., Quayle A.J. (2016). Chlamydia trachomatis Infection of Endocervical Epithelial Cells Enhances Early HIV Transmission Events. PLoS ONE.

[4] Koskela P., Anttila T., Bjørge T., Brunsvig A., Dillner J., Hakama M., Hakulinen T., Jellum E., Lehtinen M., Lenner P. (2000). Chlamydia trachomatis infection as a risk factor for invasive cervical cancer. Int. J. Cancer.

[5] Samoff E., Koumans E.H., Markowitz L.E., Sternberg M., Sawyer M.K., Swan D., Papp J.R., Black C.M., Unger E.R. (2005). Association of Chlamydia trachomatis with persistence of high-risk types of human papillomavirus in a cohort of female adolescents. Am. J. Epidemiol..

[6] Patel A.L., Chen X., Wood S.T., Stuart E.S., Arcaro K.F., Molina D.P., Petrovic S., Furdui C.M., Tsang A.W. (2014). Activation of epidermal growth factor receptor is required for Chlamydia trachomatis development. BMC Microbiol..

[7] Bastidas R.J., Elwell C.A., Engel J.N., Valdivia R.H. (2013). Chlamydial intracellular survival strategies. Cold Spring Harb. Perspect. Med..

[8] Hybiske K., Stephens R.S. (2007). Mechanisms of host cell exit by the intracellular bacterium Chlamydia. Proc. Natl. Acad. Sci. USA.

[9] Moulder J.W. (1983). Inhibition of onset of overt multiplication of Chlamydia psittaci in persistently infected mouse fibroblasts (L cells). Infect. Immun..

[10] Galasso G.J., Manire G.P. (1961). Effect of antiserum and antibiotics on persistent infection of HeLa cells with meningopneumonitis virus. J. Immunol..

[11] Poston T.B., Gottlieb S.L., Darville T. (2017). Status of vaccine research and development of vaccines for Chlamydia trachomatis infection. Vaccine.

[12] Somani J., Bhullar V.B., Workowski K.A., Farshy C.E., Black C.M. (2000). Multiple drug-resistant Chlamydia trachomatis associated with clinical treatment failure. J. Infect. Dis..

[13] Potroz M.G., Cho N.J. (2015). Natural products for the treatment of trachoma and Chlamydia trachomatis. Molecules.

[14] Saez D., Dushime R., Wu H., Ramos Cordova L.B., Shukla K., Brown-Harding H., Furdui C.M., Tsang A.W. (2019). Sulforaphane promotes chlamydial infection by suppressing mitochondrial protein oxidation and activation of complement C3. Protein Sci..

[15] Huang C.H., Chang L.C., Hu S., Hsiao C.Y., Wu S.J. (2018). Spilanthol inhibits TNFalphainduced ICAM1 expression and proinflammatory responses by inducing heme oxygenase1 expression and suppressing pJNK in HaCaT keratinocytes. Mol. Med. Rep..

[16] Huang W.-C., Wu L.-Y., Hu S., Wu S.-J. (2018). Spilanthol Inhibits COX-2 and ICAM-1 Expression via Suppression of NF-κB and MAPK Signaling in Interleukin-1β-Stimulated Human Lung Epithelial Cells. Inflammation.

[17] Hong F., Freeman M.L., Liebler D.C. (2005). Identification of sensor cysteines in human Keap1 modified by the cancer chemopreventive agent sulforaphane. Chem. Res. Toxicol..

[18] Paulraj J., Govindarajan R., Palpu P. (2013). The genus spilanthes ethnopharmacology, phytochemistry, and pharmacological properties: A review. Adv. Pharm. Sci..

[19] Spelman K., Depoix D., McCray M., Mouray E., Grellier P. (2011). The traditional medicine Spilanthes acmella, and the alkylamides spilanthol and undeca-2e-ene-8, 10-diynoic acid isobutylamide, demonstrate in vitro and in vivo antimalarial activity. Phytother. Res..

[20] Prachayasittikul V., Prachayasittikul S., Ruchirawat S., Prachayasittikul V. (2013). High therapeutic potential of Spilanthes acmella: A review. EXCLI J..

[21] Silveira N., Saar J., Santos A.D., Barison A., Sandjo L.P., Kaiser M., Schmidt T.J., Biavatti M.W. (2016). A New Alkamide with an Endoperoxide Structure from Acmella ciliata (Asteraceae) and Its in Vitro Antiplasmodial Activity. Molecules.

[22] Jagadeeswaran R., Surawska H., Krishnaswamy S., Janamanchi V., Mackinnon A.C., Seiwert T.Y., Loganathan S., Kanteti R., Reichman T., Nallasura V. (2008). Paxillin is a target for somatic mutations in lung cancer: Implications for cell growth and invasion. Cancer Res..

[23] Zheng T., Narayan R.S., Schomaker J.M., Borhan B. (2005). One-pot regio- and stereoselective cyclization of 1,2,n-triols. J. Am. Chem. Soc..

[24] Hoye T.R., Eklov B.M., Jeon J., Khoroosi M. (2006). Sequencing of three-component olefin metatheses: Total synthesis of either (+)-gigantecin or (+)-14-deoxy-9-oxygigantecin. Org. Lett..

[25] Matovic N., Matthias A., Gertsch J., Raduner S., Bone K.M., Lehmann R.P., Devoss J.J. (2007). Stereoselective synthesis, natural occurrence and CB(2) receptor binding affinities of alkylamides from herbal medicines such as *Echinacea* sp.. Org. Biomol. Chem..

[26] Nakamura A., Mimaki K., Tanigami K.I., Maegawa T. (2020). An Improved and Practical Method for Synthesizing of alpha-Sanshools and Spilanthol. Front. Chem..

[27] Julien S.G., Kim S.Y., Brunmeir R., Sinnakannu J.R., Ge X., Li H., Ma W., Yaligar J., Kn B.P., Velan S.S. (2017). Narciclasine attenuates diet-induced obesity by promoting oxidative metabolism in skeletal muscle. PLoS Biol..

[28] Holmila R.J., Vance S.A., Chen X., Wu H., Shukla K., Bharadwaj M.S., Mims J., Wary Z., Marrs G., Singh R. (2018). Mitochondria-targeted Probes for Imaging Protein Sulfenylation. Sci. Rep..

[29] Schindelin J., Arganda-Carreras I., Frise E., Kaynig V., Longair M., Pietzsch T., Preibisch S., Rueden C., Saalfeld S., Schmid B. (2012). Fiji: An open-source platform for biological-image analysis. Nat. Methods.

[30] Bolduc J.A., Nelson K.J., Haynes A.C., Lee J., Reisz J.A., Graff A.H., Clodfelter J.E., Parsonage D., Poole L.B., Furdui C.M. (2018). Novel hyperoxidation resistance motifs in 2-Cys peroxiredoxins. J. Biol. Chem..

[31] Haynes A.C., Qian J., Reisz J.A., Furdui C.M., Lowther W.T. (2013). Molecular basis for the resistance of human mitochondrial 2-Cys peroxiredoxin 3 to hyperoxidation. J. Biol. Chem..

[32] Jonsson T.J., Johnson L.C., Lowther W.T. (2008). Structure of the sulphiredoxin-peroxiredoxin complex reveals an essential repair embrace. Nature.

[33] Sullivan G.M., Feinn R. (2012). Using Effect Size-or Why the P Value Is Not Enough. J. Grad. Med. Educ..

[34] Forshaw T.E., Holmila R., Nelson K.J., Lewis J.E., Kemp M.L., Tsang A.W., Poole L.B., Lowther W.T., Furdui C.M. (2019). Peroxiredoxins in Cancer and Response to Radiation Therapies. Antioxidants.

[35] Cao Z., Roszak A.W., Gourlay L.J., Lindsay J.G., Isaacs N.W. (2005). Bovine mitochondrial peroxiredoxin III forms a two-ring catenane. Structure.

[36] Kimura A., Abe H., Tsuruta S., Chiba S., Fujii-Kuriyama Y., Sekiya T., Morita R., Yoshimura A. (2014). Aryl hydrocarbon receptor protects against bacterial infection by promoting macrophage survival and reactive oxygen species production. Int. Immunol..

[37] Subramaniam R., Barnes P.F., Fletcher K., Boggaram V., Hillberry Z., Neuenschwander P., Shams H. (2014). Protecting against post-influenza bacterial pneumonia by increasing phagocyte recruitment and ROS production. J. Infect. Dis..

[38] Azenabor A.A., Mahony J.B. (2000). Generation of reactive oxygen species and formation and membrane lipid peroxides in cells infected with Chlamydia trachomatis. Int. J. Infect. Dis..

[39] Boncompain G., Schneider B., Delevoye C., Kellermann O., Dautry-Varsat A., Subtil A. (2010). Production of reactive oxygen species is turned on and rapidly shut down in epithelial cells infected with Chlamydia trachomatis. Infect. Immun..

[40] Kading N., Kaufhold I., Muller C., Szaszak M., Shima K., Weinmaier T., Lomas R., Conesa A., Schmitt-Kopplin P., Rattei T. (2017). Growth of Chlamydia pneumoniae Is Enhanced in Cells with Impaired Mitochondrial Function. Front. Cell. Infect. Microbiol..

[41] Abdul-Sater A.A., Saïd-Sadier N., Lam V.M., Singh B., Pettengill M.A., Soares F., Tattoli I., Lipinski S., Girardin S.E., Rosenstiel P. (2010). Enhancement of reactive oxygen species production and chlamydial infection by the mitochondrial Nod-like family member, NLRX1. J. Biol. Chem..

[42] Wang X., Schwarzer C., Hybiske K., Machen T.E., Stephens R.S. (2014). Developmental stage oxidoreductive states of Chlamydia and infected host cells. mBio.

[43] Vardhan H., Bhengraj A.R., Jha R., Srivastava P., Jha H.C., Mittal A. (2010). Higher expression of ferritin protects Chlamydia trachomatis infected HeLa 229 cells from reactive oxygen species mediated cell death. Biochem. Cell. Biol..

[44] Wongsawatkul O., Prachayasittikul S., Isarankura-Na-Ayudhya C., Satayavivad J., Ruchirawat S., Prachayasittikul V. (2008). Vasorelaxant and antioxidant activities of Spilanthes acmella Murr. Int. J. Mol. Sci.

[45] Poynton R.A., Peskin A.V., Haynes A.C., Lowther W.T., Hampton M.B., Winterbourn C.C. (2016). Kinetic analysis of structural influences on the susceptibility of peroxiredoxins 2 and 3 to hyperoxidation. Biochem. J..

[46] Ali H.M., Homeida M.M., Abdeen M.A. (1988). “Drug dumping” in donations to Sudan. Lancet.

[47] Berckmans P., Dawans V., Schmets G., Vandenbergh D., Autier P. (1997). Inappropriate drug-donation practices in Bosnia and Herzegovina, 1992 to 1996. New Engl. J. Med..

[48] Gustafsson L.L., Wide K. (1981). Marketing of obsolete antibiotics in Central America. Lancet.

[49] WHO Executive Board (1994). Implementation of the global strategy for health for all by the year 2000. Second evaluation. Eighth report on the world health situation. WHO Reg. Publ. Eur. Ser..

[50] Horton R. (1996). The infected metropolis. Lancet.

[51] Korte R., Rehle T., Merkle A. (1991). Strategies to maintain health in the Third World. Tropical medicine and parasitology: Official organ of Deutsche Tropenmedizinische Gesellschaft and of Deutsche Gesellschaft fur Technische Zusammenarbeit (GTZ). Eur. PMC.

